# PCTAIRE1-Knockdown Sensitizes Cancer Cells to TNF Family Cytokines

**DOI:** 10.1371/journal.pone.0119404

**Published:** 2015-03-19

**Authors:** Teruki Yanagi, Ranxin Shi, Pedro Aza-Blanc, John C. Reed, Shu-ichi Matsuzawa

**Affiliations:** Sanford-Burnham Medical Research Institute, 10901 North Torrey Pines Road, La Jolla, California, United States of America; Innsbruck Medical University, AUSTRIA

## Abstract

While PCTAIRE1/PCTK1/Cdk16 is overexpressed in malignant cells and is crucial in tumorigenesis, its function in apoptosis remains unclear. Here we investigated the role of PCTAIRE1 in apoptosis, especially in the extrinsic cell death pathway. Gene-knockdown of *PCTAIRE1* sensitized prostate cancer PPC1 and Du145 cells, and breast cancer MDA-MB-468 cells to TNF-family cytokines, including TNF-related apoptosis-inducing ligand (TRAIL). Meanwhile, PCTAIRE1-knockdown did not sensitize non-malignant cells, including diploid fibroblasts IMR-90 and the immortalized prostate epithelial cell line 267B1. PCTAIRE1-knockdown did not up-regulate death receptor expression on the cell surface or affect caspase-8, FADD and FLIP expression levels. PCTAIRE1-knockdown did promote caspase-8 cleavage and RIPK1 degradation, while RIPK1 mRNA knockdown sensitized PPC1 cells to TNF-family cytokines. Furthermore, the kinase inhibitor SNS-032, which inhibits PCTAIRE1 kinase activity, sensitized PPC1 cells to TRAIL-induced apoptosis. Together these results suggest that PCTAIRE1 contributes to the resistance of cancer cell lines to apoptosis induced by TNF-family cytokines, which implies that PCTAIRE1 inhibitors could have synergistic effects with TNF-family cytokines for cytodestruction of cancer cells.

## Introduction

The PCTAIRE family is a branch of kinases related to the Cdk family that includes PCTAIRE1 (also known as Cyclin-dependent kinase 16 (Cdk16) and PCTK1), PCTAIRE2 and PCTAIRE3 [1]. PCTAIRE1 is broadly expressed throughout the body, with highest levels seen in the brain and testis [2]. PCTAIRE1 has been shown to participate in spermatogenesis [3] and regulation of intracellular vesicles [4,5], as well as translocation of glucose transport proteins [6] and neurite outgrowth [7]. PCTAIRE1 has a central kinase domain that shows amino acid sequence similarity to Cdks, and this region is flanked by unique N-terminal and C-terminal domains. The mechanisms responsible for PCTAIRE1 activation are unknown, but the finding that deletion of the N-terminal domain abolishes kinase activity *in vitro* implies that this region is important, and may bind an unknown cofactor or interact intra-molecularly with the central kinase domain to promote active conformations of the catalytic domain [1,7]. The N-terminal domain of PCTAIRE1 is phosphorylated by protein kinase A (PKA), which inhibits its activity [3,8], while interaction of the N-terminal domain of PCTAIRE1 with cyclin Y was shown to stimulate kinase activity [3]. PCTAIRE1 also interacts with the COPII complex involved in the export of secreted proteins from the endoplasmic reticulum [5].

We recently discovered that PCTAIRE1 plays an indispensable role in cancer cell proliferation [9,10]. We also showed that PCTAIRE1-knockdown cancer cells promoted mitotic arrest associated with defects in centrosome dynamics. Furthermore, PCTAIRE1 phosphorylates p27 at Ser10, which facilitates p27 degradation. However, the function of PCTAIRE1 in apoptosis has not been clarified.

Apoptosis induced by TRAIL, Fas-ligand (FasL) and TNF-alpha proceeds through a series of receptor-mediated protein interactions that minimally require the adapter protein FADD and cysteine proteases such as caspase-8 or-10. While these death receptor signaling complex components are retained in most cancers, resistance to apoptosis remains common. FADD and caspase-8 are among the mediators of the extrinsic pathway that are known to be modulated by protein phosphorylation, which suggests a role for kinases in resistance to pro-apoptotic TNF-family cytokines. Protein kinases are also attractive targets for cancer drug discovery. Moreover, considerable evidence has suggested a role for protein phosphorylation in modulating proximal signaling events induced by TNF-family death receptors [11–19], as well as altering the activity of well-recognized downstream apoptosis suppressors such as FLIP and Bcl-2- and IAP-family proteins [18,20–24]. In this regard, phosphorylation of the death inducing signaling complex (DISC) components Fas, FADD and caspase-8, as well as the caspase-8 substrate Bid and anti-apoptotic suppressors of death receptor-induced apoptosis (c-FLIP, XIAP) has been reported in association with tumor resistance to TRAIL or Fas [20–22,25].

In this study, we further characterized the role of PCTAIRE1 in cancer cells, and particularly its function in the extrinsic cell death pathway. We provide evidence suggesting that PCTAIRE1 plays a crucial role for resistance to TNF-family cytokines in cancer cells. Gene knockdown of *PCTAIRE1* sensitized prostate and breast cancer cells to TNF-family cytokines, including TNF-related apoptosis-inducing ligand (TRAIL) and Fas, but did not sensitize normal or non-transformed cells to TRAIL. PCTAIRE1-knockdown promoted caspase-8 cleavage and degradation of receptor-interacting serine-threonine protein kinase 1 (RIPK1). The siRNA-mediated knockdown of RIPK1 mRNA also sensitized PPC1 cells to TNF-family cytokines. Our findings suggest that PCTAIRE1 could be an important target for cancer therapy, and that PCTAIRE1 inhibitors could have synergistic effects with TNF-family cytokines to kill cancer cells.

## Materials and Methods

### Reagents and antibodies

The cell viability assay kit Cell Titer Glo was purchased from Promega. RNAiMAX was obtained from Life Technologies. Pre-designed short interfering-RNA (siRNA) directed against human PCTAIRE1 (siRNA Ids: 1472, 1566, 1656), PCTAIRE2 (s10160), PCTAIRE3 (s10162), caspase-8 (s2427), RIPK1 (s16653), p27 (s2837) and scramble-control (#1, #2) were purchased from Life Technologies. Recombinant TRAIL was purchased from Enzo Bioscience. SNS-032 was purchased from Selleck Chemicals. Antibodies against PCTAIRE1 (rabbit polyclonal: HPA001366, Sigma), RIPK1 (610458, BD Transduction Lab), caspase-8 (1C12, #9746, Cell Signaling Technology), cleaved caspase-8 (#9496, Cell Signaling Technology), phospho-caspase-8 (Ser387, #710535, Life technologies), FADD (06–711, Millipore), phospho-FADD (#2781, Cell Signaling Technology), Fas/CD95 (CH-11, SY-001, MBL), DR4 (H-130, Santa Cruz), DR5 (N-19, Santa Cruz), FLIP (F6550, Sigma), phospho-Serine (rabbit polyclonal, 61–8100, Life Technologies), p27 (mouse monoclonal G173–524: BD Pharmagen, or rabbit polyclonal C-19, Santa Cruz), HA (Rat, 3F10, Roche), HA (mouse, 12CA5, Roche), Myc (mouse, 9E10, Roche), beta-actin (mouse monoclonal, Sigma), alpha-tubulin (mouse monoclonal, B-7, Santa Cruz) and horseradish-peroxidase-conjugated secondary antibodies (GE Health Care) were purchased from the indicated sources.

### siRNA screen and data analysis

The Ambion Silencer Human Druggable Genome siRNA Library V2 was delivered into PPC1 cells by reverse transfection in 384 well format at 10 nM siRNA using RNAiMAX (Life Technologies). After 48 hours, cells were treated with 3 ng/ml anti-FAS antibody (CH-11) or DMSO for an additional 24 hours, time after which viability was read using ATP-lite (Perkin Elmer) in an Envision Plate reader (Perkin Elmer Inc.). Raw data readings were normalized to the average of negative controls included in each plate and duplicates averaged. The Effect of FAS activation was measured by calculating viability ratios CH-11/DMSO for each siRNA. A robust *Z*-score was then assigned to each siRNA. P values were also calculated for each siRNA using a 2-tail T-test assuming a normal distribution of the data. To produce a final gene score, we averaged the Z values of the 2 or 3 siRNAs targeting each gene as well as a P value using viability ratios. Values for siRNAs showing high standard deviation (> 0.25) among duplicates of either DMSO or CH-11 treated cells were removed from the analysis.

### Plasmids

Point mutations of PCTAIRE1 (K194M) was made with a PCR-based site-directed mutagenesis method using *Pfu* polymerase (Agilent). Expression plasmids for various proteins were constructed in the pcDNA3 vector for transfection or pRDI292-puro vector for lentivirus infection. Appropriate plasmid construction was confirmed by restriction enzyme digestion and DNA sequencing. The details of the primer sequences used to generate the point mutations are available upon request.

### Cell lines and cell culture

PPC1, Du145, MDA-MB-468, T47D, MCF7, IMR-90, HeLa and HEK293T cells were purchased from ATCC. 267B1 and 267B1/K-ras cells were kind gifts from Dr. Dritschilo [26]. All cells used had fewer than six months of continuous passage.

### Cell viability assays using ATP measurement

Cell Titer Glo was used for cell viability estimation. Cells were plated in 96-well solid white plates at a density of 5,000 ~ 10,000 cells per well in 100 μl complete medium with or without siRNAs and cultured for 48 hours. The cells were then treated with various concentrations of cytokines or compounds for 24 hours. Cell Titer Glo solution was added at 100 μl per well and the plates were kept in the dark for 15 minutes before measuring luminescence with a luminometer (Luminoskan Ascent; Thermo Scientific).

### Analysis of apoptosis

Cells were processed using an AnnexinV-PI apoptosis detection kit according to the manufacturer’s protocol (Life Technologies) and analyzed by flow cytometry using a FACS Canto device (Becton Dickinson).

### RNA interference

For transient knockdown, cells were transfected with siRNA duplexes by a reverse transfection method using Lipofectoamine RNAiMAX according to the manufacturer’s instructions (Life Technologies).

### Tet-inducible short hairpin RNA constructs, lentivirus and infection

PCTAIRE1 shRNA#1 (GCTCTCATCACTCCTTCACTT), PCTAIRE1 shRNA#2 (GACCTACATTAAGCTGGACAA), and scramble-control (CAACAAGATGAAGAGCACCAA) were cloned into the inducible pLKO-Tet-On puromycin vector [27]. Lentiviral supernatants were generated according to an established protocol [27]. Cells were selected with 2 μg/ml puromycin (MP Biomedicals). Induction of shRNA was achieved by the addition of 100 ng/ml doxycycline to the medium.

### Extraction of total RNA and quantitative RT-PCR analysis

Total RNA was isolated from cultured cells using the RNeasy plus mini kit (Qiagen). The RNA concentration was measured spectrophotometrically and samples were stored at-80°C until use with RT-PCR. RNA was reverse-transcribed using Superscript III (Life Technologies) and complementary DNA samples were analyzed with the SYBR green system (Promega). Primers specific for human PCTAIRE1, PCTAIRE2, PCTAIRE3, RIPK1 and control housekeeping human GAPDH are listed below.

Human PCTAIRE1

Forward: 5′- GCAGTGACCCTGGAGAGG-3′

Reverse: 5′- TCAAGTCCTCGTGCACAATC-3′

Human PCTAIRE2

Forward: 5′- TGTTATTGGAGGGAGCCTTG-3′

Reverse: 5′- TCTCACCATCTGATGCCATTT-3′

Human PCTAIRE3

Forward: 5′- ATGGCATCCACCTCCTGA-3′

Reverse: 5′- TCTGCTGACATGCGACTCTT-3′

Human RIPK1

Forward: 5′- GTGTACAAGGGGCCCAACT-3′

Reverse: 5′- CGGCTGTGTCTCAGTCTGTT-3′

Human GAPDH

Forward: 5′- GAAGGTGAAGGTCGGAGTC-3′

Reverse: 5′- ATGGGATTTCCATTGATGAC-3′

All experiments were performed in duplicate and normalized relative to GAPDH levels.

### SDS-PAGE, immunoblotting and immunoprecipitation

Cells were washed twice with PBS and harvested with radioimmunoprecipitation assay (RIPA) buffer containing 20 mM Tris-HCl, pH 7.5, 150 mM NaCl, 0.1 mM EDTA, 1% Nonidet P-40, 0.1% SDS, 5 mM NaF and EDTA-free cOmplete protease cocktail tablet (Roche). Cells were left on ice for 20 minutes and centrifuged at 14,000 x *g* for 10 minutes. Protein concentrations were measured using a Bio-Rad protein assay kit (Bio-Rad). For Laemmli SDS-PAGE, proteins were separated on SDS-PAGE 4–15% gradient gels (Life Technologies) and transferred onto nitrocellulose membranes (Bio-Rad). Phos-tag SDS-PAGE was performed with precast phostag gels (12.5% polyacrylamide gels containing 50 μM Phos-tag acrylamide, Wako). After electrophoresis, phos-tag acrylamide gels were washed with running buffer (25 mM Tris, 192 mM glycine, 0.1% SDS) containing 5 mM EDTA for 10 min with gentle shaking, and then washed again with running buffer without EDTA for 10 min according to the manufacturer’s protocol (Wako Chemical). Proteins were transferred to nitrocellulose membranes. After blocking for 1 hour in Tris-buffered saline (TBS) with 0.05% Tween-20 and 5% non-fat dry milk, membranes were incubated overnight at 4°C with primary antibodies diluted in blocking buffer. Membranes were rinsed three times in TBS with 0.05% Tween-20 and incubated with secondary HRP-conjugated antibodies for 1 hour at room temperature. An enhanced chemiluminescence (ECL) method (GE Health Care) was used for detection.

For immunoprecipitation (IP), cells were lysed in 1% NP40 buffer containing 20 mM Tris-HCl, pH 7.5, 150 mM NaCl, 0.1 mM EDTA, 1% Nonidet P-40, 5 mM NaF and EDTA-free cOmplete protease cocktail tablet. Three milligrams of protein lysate were used for immunoprecipitation with overnight incubation at 4°C and 2 μg of the given antibody. The following day, 30 μL of protein G resin (Life Technologies) was added and incubated for 1 hour at 4°C. The IPs were then washed four times with lysis buffer whereupon sample buffer was added, and the beads were boiled for 10 minutes at 100°C.

### Clonogenic assay for evaluation of Fas/TRAIL sensitivity

Cells were seeded in 35 mm dishes at 1.0 x 10^5^ cells per well and then transfected with control or PCTAIRE1-targeting siRNAs. After 2 days, agonistic anti-Fas ab (CH-11) or TRAIL was added and the cells were cultured for 3 days before fixing and staining with 0.5% crystal violet dye. For fixation, cells were incubated with methanol at-20°C for 20 minutes, washed with PBS, and incubated with 0.5% crystal violet dye in 25% methanol for 15 minutes.

### FACS analysis of Fas/DR4/DR5 expression on the cell surface

PPC1 and MDA-MB-468 cells (1.0 x 10^5^) that were reverse-transfected with siRNAs targeting PCTAIRE1 and scramble-control were seeded in 6 cm dishes. Forty-eight hours after reverse-transfection, adherent cells were washed once with PBS, detached using TripleExpress (Gibco), and resuspended in ice-cold FACS buffer (1% FBS in PBS). Following centrifugation, cells were resuspended in FACS buffer at 3.0 x 10^5^ cells/100 μl. The cells were then incubated in the dark on ice with saturating concentrations of phycoerythrin-labeled anti-DR4, anti-DR5 (eBioscience), FITC-labeled anti-Fas (BD Pharmagen), or immunoglobulin G1 isotype control antibodies for 1 hour as per the manufacturer’s instructions. A total of 10,000 events were analyzed by flow cytometry for each treatment.

### Synchronization

To arrest HeLa cells in early S phase, double thymidine block method was applied [10]. Briefly, thymidine (2.5 mM) was added to culture for 14 hours, and cells were released from the block by washing three times with PBS. After 8 hours, thymidine was added again. After a second treatment for 14 hours, cells were released from the block.

### Quantitative Measurement and Statistical Analysis

Means and standard deviation (SD) were calculated statistically from three determinations. The data are expressed as mean ± SD. Statistical significance of differences between various samples was determined by Student’s t-test. P < 0.05 was considered significant.

## Results

### Identification of PCTAIRE1 kinase by siRNA library screening

To identify candidate suppressors or enhancers of Fas-induced apoptosis in tumor cells, we optimized two types of high throughput screening (HTS) assays for siRNA library screening. Using PPC1 prostate cancer cells, we determined that stimulation with low dose anti-Fas antibody CH11 (3 ng/ml) did not result in cytotoxicity, while stimulation with high dose anti-Fas antibody (25 ng/ml) killed these tumor cells. Using control siRNAs to validate the assay, we determined that 3 of 3 siRNAs targeting FLIP sensitized PPC1 cells to low-dose (3 ng/ml) anti-Fas antibody whereas 3 of 3 siRNAs targeting FADD inhibited PPC1 killing induced by high-dose (25 ng/ml) anti-Fas antibody. After using these control siRNAs to optimize the HTS assays to a Z’ factor > 0.5, we then screened a library of 16,800 siRNAs targeting 5,600 genes (3-fold coverage), which constitutes the so-called “druggable genome”. PPC1 cells in 384 well plates were “reverse” transfected with siRNAs, then 2 days later CH11 antibody was added and cell viability assessed using a bioluminescence assay to determine ATP levels one day later (S1 Fig., S1 Dataset). The effect of FAS activation was measured by calculating viability ratios Fas/DMSO for each siRNA. To produce a final gene score, we averaged the Z values of the 2 or 3 siRNAs targeting each gene. For the low-dose (3 ng/ml) screen, the PCTAIRE1 kinase was identified as a suppressor of Fas-induced apoptosis.

### Gene knockdown of PCTAIRE1 sensitizes cancer cells to TNF-family cytokines

**Fig 1:**
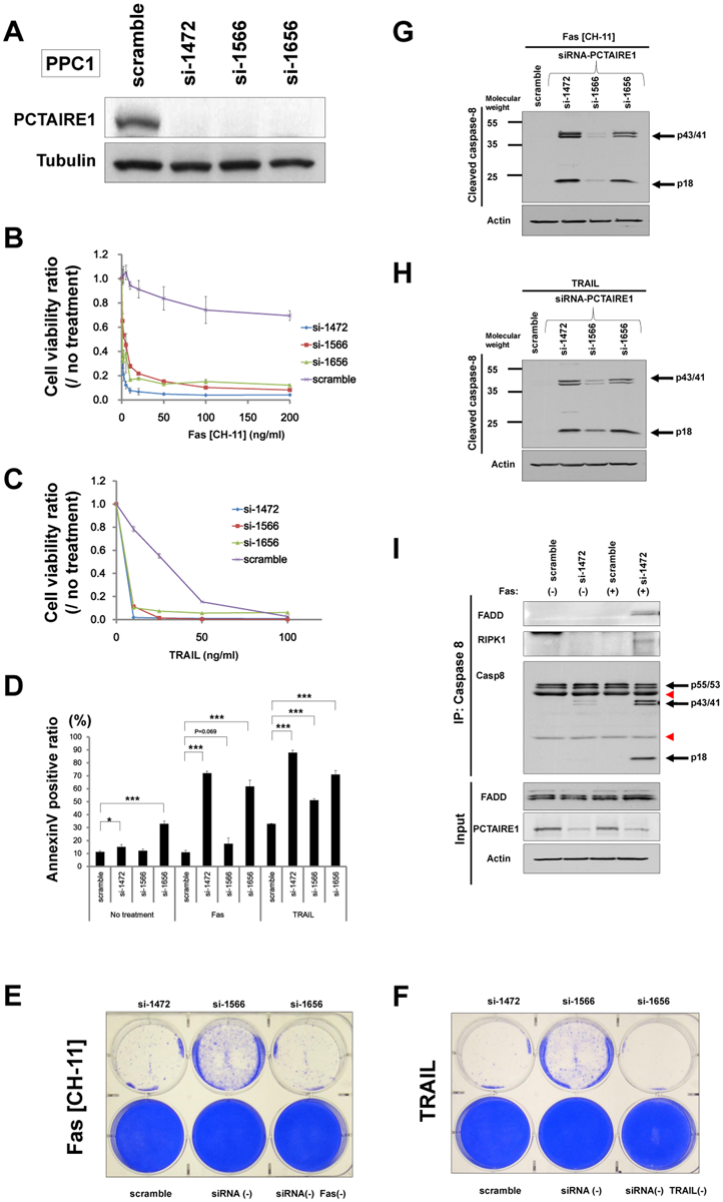
PCTAIRE1 knockdown sensitizes PPC1 cells to anti-Fas antibody and TRAIL. (A) PPC1 cells were transfected with scrambled RNA or three different siRNAs targeting PCTAIRE1 (siRNAs 1472, 1566, 1656). Lysates from cells at 48 hours post-siRNA transfection were prepared, normalized for total protein content, and aliquots were analyzed by immunoblotting using anti-PCTAIRE1 (top) or anti-alpha-tubulin (bottom) antibodies. (B, C) PPC1 cells were transfected with control RNA (purple “x”) or various siRNAs targeting PCTAIRE1 (blue diamonds, 1472; red squares, 1566, green triangles, 1656). After 48 hours, cells were stimulated with either anti-Fas antibody (CH-11) (B) or TRAIL (C) at various concentrations as indicated. After 24 hours, cellular ATP levels were measured as a surrogate indicator of cell viability using Cell Titer Glo reagents, and the data are expressed as the ratio between cells cultured with and without anti-Fas (B), and TRAIL (C). (D) Apoptosis analyses were performed using an annexinV kit. PPC1 cells were transfected with scramble RNA of siRNAs targeting PCTAIRE1. After 48 hours, cells were treated with anti-Fas antibody (100 ng/ml) or TRAIL (50 ng/ml) for 4 hours. *P < 0.05, ***P < 0.001 by t-test. All data represent mean ± SD (n = 3). (E, F) Clonogenic survival assays. PPC1 cells were seeded in 6 well (35 mm) dishes at 1.0 x 10^5^ cells per well and then reverse-transfected with scramble-control or PCTAIRE1-targeting siRNAs as indicated. After 48 hours, anti-Fas antibody (E, 10 ng/ml) or TRAIL (F, 20 ng/ml) was added and cells were cultured for 3 days before fixing and staining with 0.5% crystal violet dye. (G, H) PPC1 cells were transfected with control RNA or three different siRNAs targeting PCTAIRE1. After 48 hours, the cells were stimulated with Fas (G, 10 ng/ml) or TRAIL (H, 10 ng/ml). After 3 hours, cell lysates were prepared, normalized for total protein content, and analyzed by immunoblotting using an antibody specific for cleaved caspase-8. The partially cleaved 43/41 kDa bands and fully cleaved 18 kDa band are indicated, as are molecular weight markers (kDa). The blot was reprobed with beta-actin antibody as a loading control. (I) PPC1 cells were transfected with scramble-control or siRNA targeting PCTAIRE1 (si-1472). After 48 hours, cells were or were not stimulated with anti-Fas antibody (CH-11, 10 ng/ml). After 3 hours, cell lysates were harvested and immunoprecipitated with anti-caspase 8 antibody, followed by immunoblotting with the indicated antibodies. The red arrowheads indicate non-specific bands.

To further assess the role of PCTAIRE1 on TNF-family cytokines in prostate cancer PPC1 cells, we used siRNAs targeting PCTAIRE1. Immunoblotting was first performed to confirm knockdown at the protein level (Fig. 1A). Using 3 siRNAs that successfully knocked down PCTAIRE1 expression, we observed sensitization of PPC1 cells to anti-Fas antibody (CH-11), TRAIL, and TNF-alpha (Fig. 1B and C, and S2A Fig.). AnnexinV staining experiments independently confirmed these results, and provided evidence that an apoptotic mechanism was involved (Fig. 1D). In contrast to Fas (CH-11), TRAIL and TNF, PCTAIRE1 knockdown did not sensitize PPC1 cells to other cell death pathways, including stimuli that initiate apoptosis pathways from the endoplasmic reticulum (thapsigargin) and mitochondria (staurosporine) (S2B Fig. and data not shown). Similar conclusions were reached when clonogenic survival was assessed, with all 3 siRNAs targeting PCTAIRE1 sensitizing PPC1 cells to Fas (CH-11) and TRAIL (Fig. 1E and F). In contrast, siRNA-mediated knockdown of PCTAIRE2 or PCTAIRE3 did not sensitize PPC1 cells to Fas, TRAIL or TNF (S2C-H Fig.). We also assessed the effect of PCTAIRE1-knockdown on the chemosensitivity of cells to cisplatin and paclitaxel. PPC1 cells with PCTAIRE1-knockdown were incubated with different concentrations of cisplatin and paclitaxel, and assessment of cell viability showed no significant synergistic cytotoxic effects in PPC1 cells (S2I-N Fig.). We further assessed siRNA-mediated PCTAIRE1-knockdown in other cell lines, and found that in breast cancer MDA-MB-468 cells PCTAIRE1-knockdown was effective at restoring sensitivity to Fas or TRAIL (S3A-F Fig.). However, as would be expected for the heterogeneity seen in human cancers, the effect of PCTAIRE1 knockdown was not uniform in that less robust effects were observed with human breast cancer MCF7 cells (S3G-I Fig.).

The activation of caspase-8 induced by TNF-family death receptors is accompanied by proteolytic processing of the ~50 kDa pro-caspase-8 molecule initially to yield ~43/41 kDa partially processed molecules and finally to produce the ~18 and ~10 kDa subunits of the catalytically active protease. We used immunoblotting to monitor the effects of siRNA-mediated knockdown of PCTAIRE1 on the proteolytic processing of pro-caspase-8. The siRNA-mediated silencing of PCTAIRE1 in PPC1 cells indeed increased Fas- and TRAIL-induced processing of caspase-8 compared to control RNA (Fig. 1G and H). We also observed the assembly of the FADD/caspase-8 complex (death-inducing signaling complex: DISC) in Fas-treated PPC1 cells with PCTAIRE1 knockdown (Fig. 1I). Conversely, knockdown of caspase-8 confered Fas-resistance on PPC1 cells with PCTAIRE1-knockdown (data not shown).

**Fig 2:**
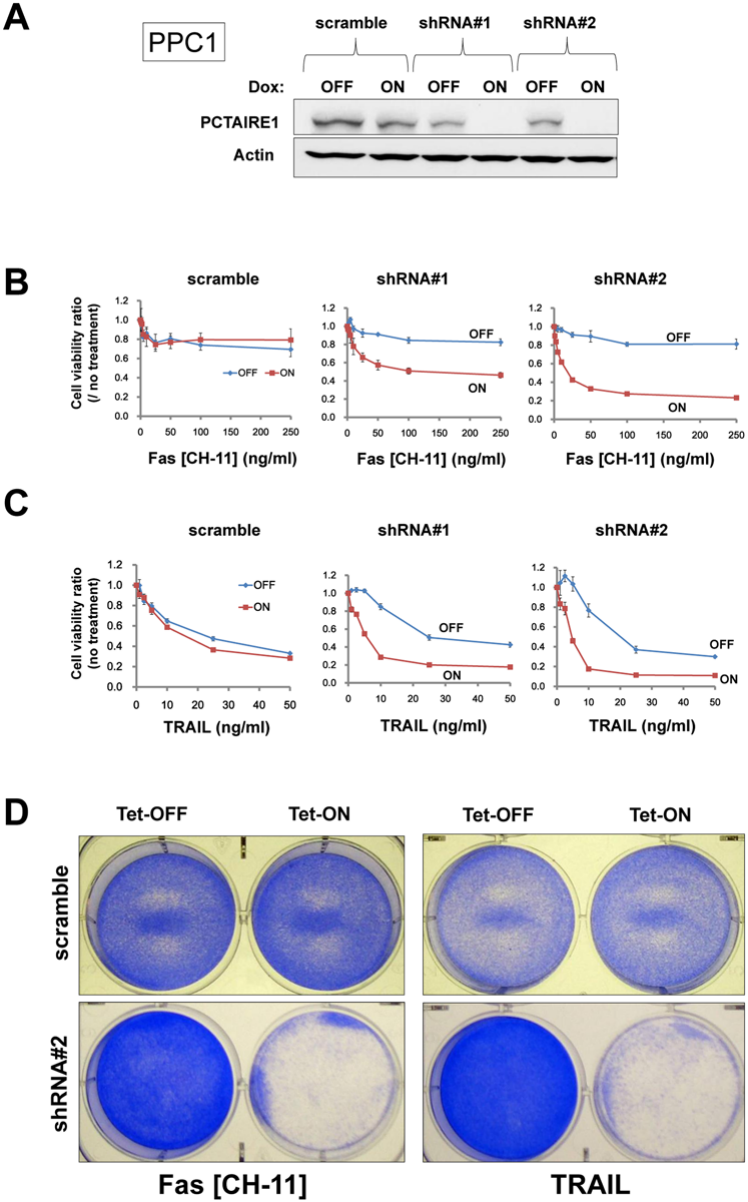
Targeting PCTAIRE1 using an inducible shRNA vector in PPC1 cells. (A) PPC1 cells stably containing inducible shRNAs targeting different sites on PCTAIRE1 mRNA (shRNA#1, #2) or scramble-control were cultured for 48 hours with doxycycline (Dox, 100 ng/ml). Protein lysates were generated, normalized for total protein concentration, and analyzed by SDS-PAGE/immunoblotting using antibodies for PCTAIRE1 (top) and beta-actin (bottom). (B, C) PPC1 cells were cultured with (ON) or without (OFF) 100 ng/ml Dox for 48 hours, then stimulated with various concentrations of either anti-Fas antibody CH-11 (B) or TRAIL (C). After 24 hours, cellular ATP levels were measured, and the data expressed as a ratio relative to cells cultured without anti-Fas or TRAIL (mean ± SD; n = 3). (D, E) Clonogenic survival assays. PPC1 cells stably containing inducible shRNAs (scramble or shRNA#2) were cultured with (ON) or without (OFF) 100 ng/ml Dox for 48 hours, then stimulated with anti-Fas antibody (CH-11, 10 ng/ml) or TRAIL (20 ng/ml). Cells were cultured for 3 days before fixing and staining with 0.5% crystal violet dye.

The siRNA-mediated PCTAIRE1-knockdown effects on the extrinsic cell death pathway were also reproduced using tetracycline-inducible shRNA vectors introduced by lentivirus infection [27]. In PPC1 cells that were stably infected with two different shRNAs targeting PCTAIRE1 (shRNA#1 and #2), culturing with the tetracycline analog doxycycline resulted in reduced PCTAIRE1 protein levels (Fig. 2A). Correlating with these reductions in the PCTAIRE1 protein, doxycycline also restored PPC1 sensitivity to Fas and TRAIL, as indicated by cell viability assay results (Fig. 2B-E). Similar results were obtained with two other tumor cell lines (MDA-MB-468 and Du145 cells) that were stably transduced with tetracycline-inducible PCTAIRE1 shRNA vectors (S4 and S5 Figs.). From these results, we conclude that PCTAIRE1 confers Fas/TRAIL resistance on epithelial cancer cells.

### Transformed cells are preferentially dependent on PCTAIRE1 for resistance to TRAIL

**Fig 3:**
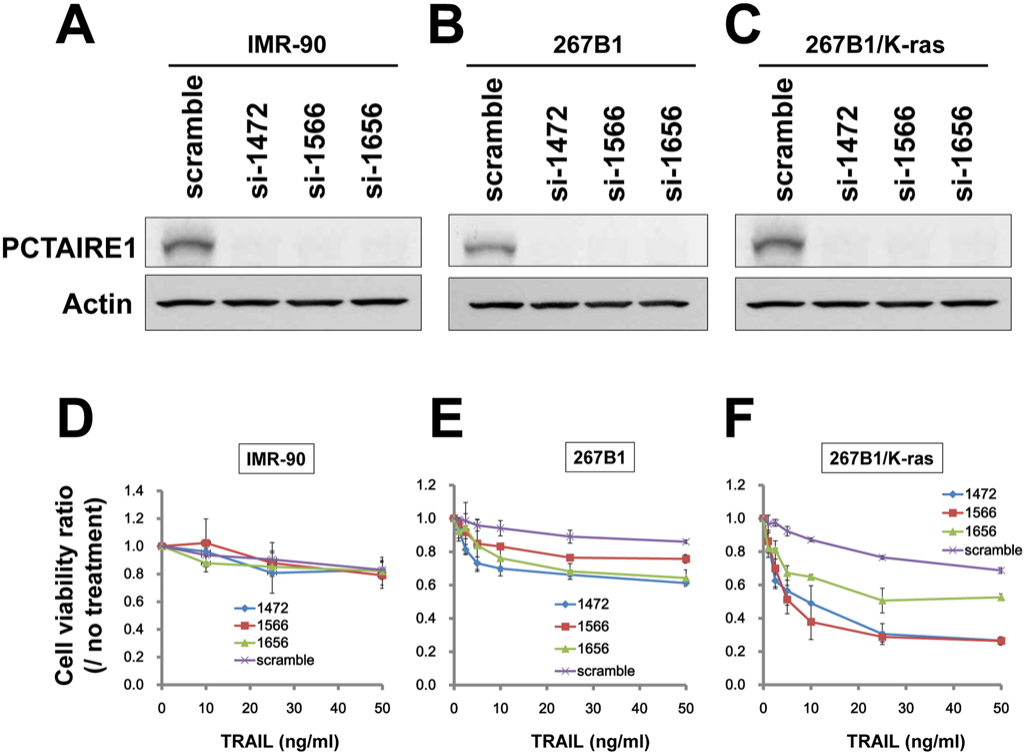
Transformed cells are preferentially sensitive to PCTAIRE1 knockdown-effect with TRAIL treatment. (A-C) Human diploid fibroblasts IMR-90 (A), 267B1 immortalized normal prostate epithelial cells (B), and K-ras transformed 267B1 (267B1/K-ras) cells (C) were transfected with scrambled RNA or three different siRNAs targeting PCTAIRE1 (siRNAs: si-1472, si-1566, si-1656). Lysates of cells 72 hours post-transfection were prepared and analyzed by immunoblotting using rabbit anti-PCTAIRE1 (top) or anti-beta-actin (bottom) antibodies. (D-F) IMR-90, 267B1 and 267B1/K-ras cells were transfected with siRNAs. After 48 hours, cells were stimulated with TRAIL at various concentrations as indicated. After 24 hours, cellular ATP levels were measured, and the data expressed as a ratio between cells cultured with and without TRAIL (mean ± SD; n = 3).

Among the TNF-family cytokines, TRAIL is considered the most promising weapon for combating cancer cells because TRAIL sensitivity is observed predominantly in tumor but not normal cells [28,29]. Furthermore, histopathological analyses of primary prostate and breast tissues revealed that PCTAIRE1 immunostaining was very low in normal tissues, but markedly higher in cancers [10]. These observations suggest that PCTAIRE1-knockdown would not affect the extrinsic cell death pathway in normal cells. To support this possibility, we examined the knockdown of PCTAIRE1 in the human diploid fibroblast line IMR-90. In these cells siRNA-mediated PCTAIRE1-knockdown did not affect TRAIL sensitivity as assessed by measuring ATP levels (Fig. 3A and D). To further examine the role of PCTAIRE1 in normal versus transformed cells, we applied PCTAIRE1-siRNAs to immortalized 267B1 normal prostate epithelial cells and their K-ras (V12) transformed isogenic counterpart, 267B1/K-ras cells [26], followed by confirmation of siRNA efficacy using immunoblotting (Fig. 3B and C). Sensitivity to TRAIL was assessed by ATP levels, which indicated that 267B1/K-ras cells with PCTAIRE1 knockdown were remarkably sensitive to TRAIL compared to control 267B1/K-ras cells with scramble-siRNA (Fig. 3F). In contrast, non-transformed 267B1 cells with PCTAIRE1-knockdown showed no significant change in TRAIL sensitivity compared to scramble-control cells (Fig. 3E). These results suggest that the transformed cells are preferentially dependent on PCTAIRE1 for resistance to TRAIL.

### PCTAIRE1-knockdown modulates RIPK1 protein expression

**Fig 4:**
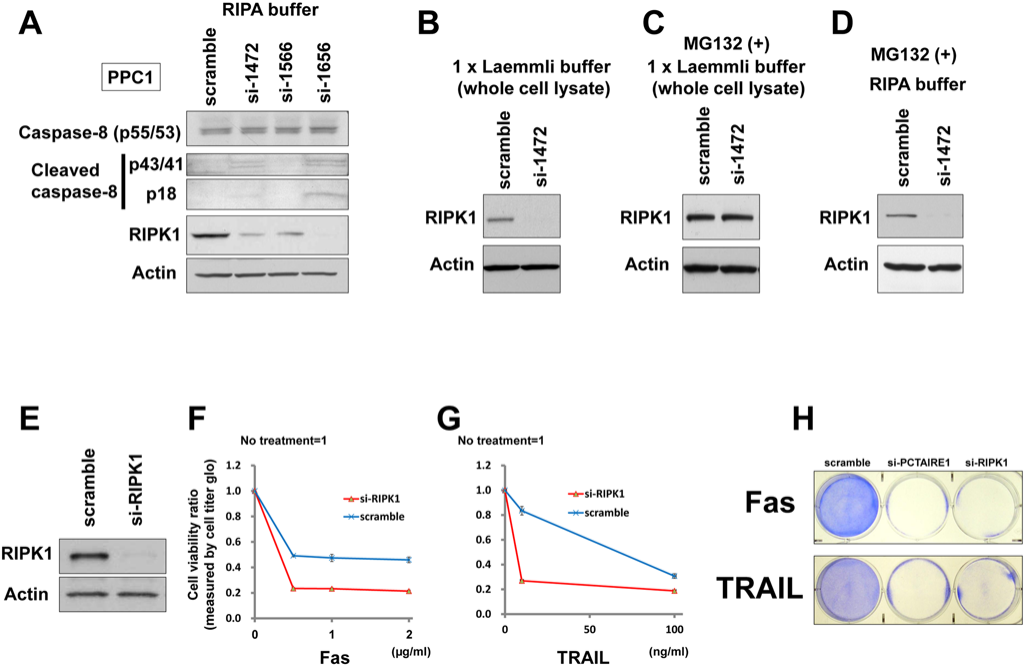
PCTAIRE1-knockdown modulates RIPK1 expression. (A) PPC1 cells were transfected with control RNA or three different siRNAs targeting PCTAIRE1. After 48 hours, cell lysates were prepared with RIPA buffer, normalized for total protein content, and analyzed by immunoblotting using the indicated antibodies. (B) PPC1 cells were transfected with indicated siRNAs for 48 hours, whereupon cell lysates were collected in 1x Laemmli buffer and subjected to immunoblotting analysis for RIPK1 (top) and beta-actin (middle). (C, D) PPC1 cells were transfected with the indicated siRNAs. After 48 hours, cells were treated with the proteasome inhibitor MG-132 (10 μM) for 5 hours before preparation of cell lysates (C: 1x Laemmli buffer, D: RIPA buffer). (E) PPC1 cells were transfected with control RNA or siRNA targeting RIPK1. After 48 hours, cell lysates were prepared in RIPA buffer, normalized for total protein content, and analyzed by immunoblotting using the indicated antibodies. (F, G) PPC1 cells were transfected with control RNA or siRNA targeting RIPK1. After 48 hours, cells were stimulated with either anti-Fas antibody (CH-11) (F) or TRAIL (G) at various concentrations as indicated. After 24 hours, cellular ATP levels were measured as a surrogate indicator of cell viability using Cell Titer Glo reagents, and the data expressed as a ratio between cells cultured with and without anti-Fas (F) or TRAIL (G). (H) Clonogenic survival assays. PPC1 cells were seeded in 6 well (35 mm) dishes at 1.0 x 10^5^ cells per well, and then reverse-transfected with scramble-control, PCTAIRE1-targeting siRNA (si-1472), or RIPK1-targeting siRNA as indicated. After 48 hours, anti-Fas antibody (top, 2.5 ng/ml) or TRAIL (bottom, 10 ng/ml) was added and cells were cultured for 3 days before fixing and staining with 0.5% crystal violet dye.

To explore the mechanism by which PCTAIRE1 suppresses apoptosis induced by TRAIL/Fas, we examined the impact of PCTAIRE1 knockdown on expression of various proteins that have been implicated in the TNF-family cytokine receptor signaling pathway. Because PCTAIRE1-knockdown sensitized PPC1 cells to death receptor stimuli (Fas, TRAIL) but not agents that trigger other cell death pathways, we suspected that PCTAIRE1 may block a proximal step in Fas and TRAIL receptor signaling. The critical event for initiating the apoptosis pathway induced by TNF-family cytokines is activation of caspase-8, which requires the adapter protein FADD, and is opposed by FLIP. As shown above, PCTAIRE1-knockdown activated caspase-8 processing (Fig. 4A), but PCTAIRE1-knockdown did not affect the expression levels of FADD, procaspase-8 or c-FLIP (S6A Fig.). Measurement of cell surface levels (by FACS) or total cellular levels (by immunoblotting) of Fas, TRAIL-receptor 1 (death receptor 4, DR4), or TRAIL-receptor 2 (death receptor 5, DR5) also showed no reproducible difference between control and PCTAIRE1-knockdown cells (S6A and B Fig., and data not shown). Moreover, while phosphorylation of caspase-8 and FADD are reportedly involved in resistance to TNF family cytokines [11,12,30], we detected no remarkable change in the phosphorylation levels of caspase-8 (Ser387) or FADD (Ser194) in PCTAIRE1 overexpressing/knockdown PPC1 cells (S6C-F Fig.).

Next, we used immunoblotting to assess RIPK1 expression in PCTAIRE1-knockdown cells and found that RIPK1 levels in PCTAIRE1-knockdown PPC1 cells were lower than control cells (Fig. 4A). However, RIPK1 mRNA levels were not down-regulated in PCTAIRE1-knockdown PPC1 cells compared to control cells (S7A Fig.). Down-regulation of RIPK1 protein levels by PCTAIRE1-knockdown was also seen in MDA-MB-468 cells (S7B and C Fig.). Meanwhile, PCTAIRE1-knockdown did not induce similar RIPK1 downregulation in MCF7 cells (S7D and E Fig.).

Because PCTAIRE1 knockdown caused a decrease in RIPK1 protein levels without impacting mRNA levels, we hypothesized an effect on RIPK1 protein stability. To this end, the effect of PCTAIRE1 on RIPK1 protein stability was examined by immunoblotting of cell lysates treated with the proteasome inhibitor MG132, which reduced PCTAIRE1-knockdown-mediated RIPK1 degradation (Fig. 4B and C). To examine whether the expression of RIPK1 is dependent on cell cycle phase, we assessed RIPK1 protein levels from early S to M phases by cell cycle synchronization experiments, which showed that RIPK1 protein levels were stable during S-M phases (S7F Fig.). Furthermore, when cells were collected in RIPA buffer and only soluble protein extracts were used for immunoblotting, we detected no RIPK1 recovery from PCTAIRE1-knockdown cells in the presence of MG132 (Fig. 4D), suggesting that PCTAIRE1 knockdown causes RIPK1 to partition largely into the insoluble fraction.

Since RIPK1 degradation or deficiency has been reported to sensitize cells to TNF-induced apoptosis [31–33], we also assessed RIPK1-knockdown effects on TNF-family mediated apoptosis. Using siRNA that successfully knocked down RIPK1, we observed sensitization of PPC1 cells to anti-Fas antibody (CH-11) and TRAIL (Fig. 4E-H). We next analyzed the effects of the necrostatin-1 (RIPK1-inhibitor) on TRAIL-induced cell death. Preincubation with the necrostatin-1 showed neither reduced nor enhanced effects of TRAIL on cell death (S7G Fig.). Furthermore, we assessed phosphorylation status of RIPK1 in PCTAIRE1-knockdown PPC1 cells by phos-tag SDS-PAGE analysis, but did not detect a discernible change of RIPK1 phosphorylation upon PCTAIRE1-knockdown (S7H Fig.). These results suggest that sensitization to death receptors caused by decreased RIPK1 is a dependence of a protein scaffold function rather than an enzymatic kinase activity of RIPK1. Furthermore, in overexpression experiments PCTAIRE1 did not affect RIPK1 phosphorylation (data not shown). To examine the interaction between PCTAIRE1 and RIPK1, we performed an immunoprecipitation analysis and a yeast two hybrid assay. However, no direct interaction between these proteins was observed in these assays (data not shown). Together, these results suggest that low expression of RIPK1 in PCTAIRE1 knockdown cancer cells potentially explains the sensitization to TNF family cytokines, but the mechanism is unclear and alternative explanations may exist.

Our previous study revealed that PCTAIRE1 phosphorylates p27 and promotes its degradation [10]. To investigate the role of accumulated p27 on Fas/TRAIL-sensitivity, PPC1 cells with Tet-inducible shRNA (#2) were transfected with siRNAs targeting p27 or scramble-control. Immunoblot analysis of the transfected cells confirmed PCTAIRE1 and p27 double-knockdown two days after transfection (S8A Fig.). At 24 hours after Fas or TRAIL treatment, no significant difference in cell viability was observed between p27 and PCTAIRE1 double-knockdown PPC1 cells versus PCTAIRE1 single-knockdown PPC1 cells (S8B and C Fig.). We also assessed TRAIL-sensitivity in PPC1 cells with lentivirus-mediated p27 overexpression and found no significant difference in cell viability between PPC1 cells with or without p27 overexpression 24 hours after TRAIL treatment (S8D and E Fig.). These results suggest that p27 accumulation does not account for Fas/TRAIL-sensitivity observed in PCTAIRE1-knockdown cells.

### SNS-032 sensitizes PPC1 cells to TRAIL-induced apoptosis

**Fig 5:**
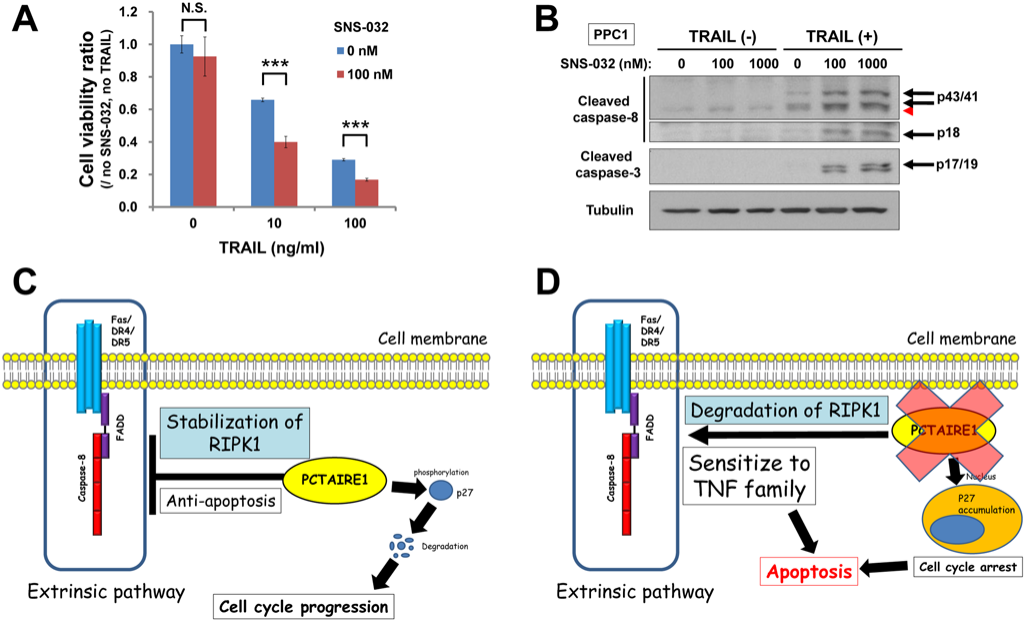
SNS-032 synergizes with TRAIL to kill PPC1 cells. (A) PPC1 cells were preincubated with DMSO or SNS-032 (100 nM) for 3 hours and subsequently stimulated with TRAIL (0, 10, 100 ng/ml). Cell viability was determined after 24 hours. N.S., not significant, ***P < 0.001 by t-test. All data represent mean ± SD (n = 3). (B) PPC1 cells were preincubated with DMSO or SNS-032 (100 nM or 1,000 nM) for 3 hours and subsequently stimulated with TRAIL (0 or 100 ng/ ml) for 3 hours. Cells were lysed and subjected to immunoblotting. A red arrowhead indicates a non-specific band. (C, D) Model of PCTAIRE1 function. (C) PCTAIRE1 contributes to the resistance of cancer cell lines to killing by TNF-family cytokines (extrinsic cell death pathway) via stabilization of RIPK1, which is independent from p27 phosphorylation. (D) PCTAIRE1-knockdown promoted RIPK1 degradation, and sensitized cancer cells to TNF-family cytokines.

In our previous study, we showed that the kinase inhibitor SNS-032 suppresses PCTAIRE1 kinase activity [10]. Based on that study, we evaluated the effects of a combination therapy of SNS-032 and TRAIL in cancer cells. PPC1 cells treated with SNS-032 showed marked sensitization to TRAIL-induced cell death (Fig. 5A). Meanwhile, cells treated with SNS-032 and TRAIL together showed increased caspase-8 and caspase-3 cleavage (Fig. 5B), consistent with an apoptotic mechanism Our results are consistent with the study by Lemke *et al*., which reported that SNS-032 sensitizes cancer cells to TRAIL [34]. Our studies also suggest the possibility that one mechanism explaining SNS-032-induced sensitization to TRAIL may be inhibition of PCTAIRE1 activity, thus suggesting that more selective inhibitors of this kinase should be generated for further exploring this hypothesis.

## Discussion

In this study we report a role for the PCTAIRE family member PCTAIRE1 in modulating the extrinsic cell death pathway (Fig. 5C and D). Gene knockdown of *PCTAIRE1* sensitized several prostate and breast cancer cells to TNF-family cytokines, whereas PCTAIRE1-knockdown did not sensitize normal or non-transformed cells to TRAIL.

Our study showed that the protein kinase PCTAIRE1 is involved in the extrinsic cell death pathway. To date, the relationship between Cdk-family proteins and the extrinsic cell death pathway has not been characterized in detail. Cdk1 has been reported to phosphorylate caspase-8 at Ser 387, which confers TNF-family resistance to HeLa cells [30]. Since PCTAIRE1 shares amino acid sequence similarity with Cdk1 [1], PCTAIRE1 might be expected to phosphorylate caspase-8 as well, although we observed no obvious up-regulation of caspase-8 phosphorylation at the same residue using phostag SDS-PAGE (data not shown). However, we cannot exclude the possibility that a subpopulation of caspase-8 molecules undergoes changes in protein phosphorylation that require more sensitive methods for detection. Recently, Lemke *et al*. reported that inhibition of Cdk9 overcomes TRAIL resistance via suppression of Mcl-1 and FLIP protein expression [34]. They also showed the preclinical efficacy of combining the “Cdk-inhibitor” SNS-032 with TRAIL. Based on that study, we assessed Mcl-1 and FLIP expression in PCTAIRE1 knockdown cells, but the expression levels of these proteins were not changed substantially (S3 Fig., and data not shown).

Our results suggested that RIPK1 is associated with activation of the extrinsic cell death pathway in PCTAIRE1 knockdown cancer cells, which supports findings in several reports indicating that modulation of RIPK1 expression is associated with the extrinsic apoptotic pathway. Furthermore, disruption of heat shock protein 90 (Hsp90) function by geldanamycin causes RIPK1 degradation, which sensitizes tumor cells to TNF-induced apoptosis [31]. Another report showed that treatment of human breast cancer cells with the Hsp90 inhibitor 17DMAG (17-dimethylaminoethylamino-17-demethoxygeldanamycin) facilitated TRAIL-induced apoptosis, and that RNAi-mediated gene knockdown of RIPK1 is sufficient to sensitize human breast tumor cells to TRAIL-induced apoptosis [32]. Together these findings are largely consistent with our results implicating RIPK in the mechanism by which PCTAIRE1 modulates TRAIL sensitivity. Moreover, we were unable to uncover the mechanism by which PCTAIRE1 regulates RIPK1 stability, failing to find changes in phosphorylation or interaction of these proteins. Interestingly, our observation that siRNA-mediated knockdown of RIPK1 but not RIPK1 inhibition by a small molecule suggests that RIPK1’s role in suppressing death receptor-mediated tumor cell killing is not due to its kinase activity but rather perhaps due to its participation in critical protein-protein interactions.

In our previous study, tumor cells with PCTAIRE1 knockdown show late G2-M phase arrest [10]. To assess the effect of cell cycle arrest in the sensitization to Fas/TRAIL, we performed siRNA experiments targeting Cdk1. As expected, prostate cancer PPC1 cells subjected to Cdk1 knockdown showed G2-M phase arrest; however, Cdk1 knockdown did not sensitize PPC1 cells to Fas or TRAIL (data not shown). Thus, cell cycle arrest by PCTAIRE1 knockdown does not seem to sensitize tumor cells to Fas/TRAIL. Further, we investigated the cell cycle dependence of RIPK1 expression using synchronized HeLa cells. The RIPK1 protein levels were stable during early S-M phases of cell cycle; thus, down regulation of RIPK1 by PCTAIRE1-knockdown is not thought to be associated with cell cycle arrest.

In conclusion, we identified PCTAIRE1 as a contributor to resistance of cancer cell lines to killing by TNF-family cytokines. Although the mechanism of PCTAIRE1 awaits additional characterization, the results presented here suggest the potential of PCTAIRE1 as a possible target for cancer therapy. In this regard, for patients with metastatic cancers, a need exists for more effective and less toxic therapies that improve survival. Recently, immune-based therapies for cancers have attracted great interest, showing promising potential for producing long-term remissions for some patients [35]. In as much as TNF-family cytokines are among the weapons used by cytolytic T-cells (CTLs) and Natural Killer (NK) cells to eradicate tumor cells, inhibitors of PCTAIRE1 could be considered for sensitizing to cancer immunotherapeutics such as immune checkpoint modulators (PD-1, PD-L1, CTLA4) and chimeric antigen receptor (CAR) T-cells.

## Supporting Information

S1 Dataset siRNA screen and data analysis. (XLSX)

S1 Fig Strategy for siRNA library screening. Two parallel HTS campaigns were conducted using low-dose (3 ng/ml) and high-dose (25 ng/ml) anti-Fas CH11 antibody. A library of 16,800 siRNAs constituting the “druggable” genome of 5,600 targets at 3-fold coverage was screened.
(TIF)

S2 Fig PCTAIRE1 knockdown does not sensitize PPC1cells to thapsigargin, cisplatin or paclitaxel. (A, B) PPC1 cells were transfected with control RNA (purple “x”) or various siRNAs targeting PCTAIRE1 (blue diamonds, 1472; red squares, 1566, green triangles, 1656). After 48 hours, cells were stimulated with either TNF (A) or thapsigargin (B) at the indicated concentrations. After 24 hours, cellular ATP levels were measured and the data expressed as the ratio between values for cells cultured with and without TNF (A) and thapsigargin (B) (mean ± SD; n = 3). (C-H) PPC1 cells were transfected with scrambled RNA or siRNAs targeting PCTAIRE1 (si-1472), PCTAIRE2 or PCTAIRE3. At 48 hours after transfection, mRNAs levels of PCTAIRE1 (C), PCTAIRE2 (D) and PCTAIRE3 (E) were measured by qRT-PCR, with normalization relative to GADPH (mean ± SD; n = 2). Forty-eight hours after transfection, cells were stimulated with Fas (CH-11) (F), TRAIL (G), or TNF (H) at various concentrations as indicated. After 24 hours, cellular ATP levels were measured, and the data expressed as the ratio between values for cells cultured with and without treatments (mean ± SD; n = 3). (I-N) PPC1 cells stably containing inducible shRNAs targeting different sites on PCTAIRE1 mRNA (shRNA#1, #2) or scramble-control were cultured for 48 hours with doxycycline (Dox, 100 ng/ml). PPC1 cells were cultured with (ON) or without (OFF) 100 ng/ml Dox for 48 hours, then stimulated with various concentrations of either cisplatin (I-K) or paclitaxel (L-N). After 24 hours, cellular ATP levels were measured and expressed as a ratio relative to cells cultured without treatments (mean ± SD; n = 3).
(TIF)

S3 Fig PCTAIRE1 knockdown sensitizes MDA-MB-468 cells to anti-Fas antibody and TRAIL. (A, G) MDA-MB-468 (A) and MCF7 (G) cells were transfected with scrambled RNA or three different siRNA targeting PCTAIRE1 (siRNAs 1472, 1566, 1656). After 48 hours, relative levels of PCTAIRE1 mRNA were measured by q-RT-PCR analysis. (B) Cell lysates 48 hours after transfection of siRNAs were prepared, normalized for total protein content, and aliquots were analyzed by immunoblotting using mouse anti-PCTAIRE1 (top) or anti-beta-actin (bottom) antibodies. (C, D, H, I) MDA-MB-468 (C, D) and MCF7 (H, I) cells were transfected with control RNA (purple “x”) or various siRNAs targeting PCTAIRE1 (blue diamonds, 1472; red squares, 1566; green triangles, 1656). After 48 hours, cells were stimulated with either anti-Fas antibody or TRAIL at various concentrations as indicated. After 24 hours, cellular ATP levels were measured and the data expressed as the ratio between values for cells cultured with and without anti-Fas (C, H) or TRAIL (D, I). All data represent mean ± SD (n = 3). (E, F) Clonogenic survival assays show that PCTAIRE1-knockdown sensitizes MDA-MB-468 cells to Fas and TRAIL. MDA-MB-468 cells were seeded at 2.0 x 10^5^ cells per well in 6 well (35 mm) dishes, then reverse-transfected with control or PCTAIRE1-targeting siRNAs as indicated. After 48 hours, anti-Fas antibody (250 ng/ml) (E) or TRAIL (100 ng/ml) (F) was added and cells were cultured for 72 hours before fixing and staining with 0.5% crystal violet dye.
(TIF)

S4 Fig Targeting PCTAIRE1 using an inducible shRNA vector in MDA-MB-468 cells. (A) MDA-MB-468 cells stably expressing inducible shRNAs targeting different sites on PCTAIRE1 mRNA (shRNA#1, #2) were cultured for 48 hours with various concentrations of doxycycline (Dox) ranging from 10 to 1000 ng/ml. PCTAIRE1 mRNA levels were measured by qRT-PCR, with normalization relative to GADPH (mean ± SD; n = 2). (B) Protein lysates were generated from MDA-MB-468 cells cultured for 48 hours with (ON) or without (OFF) 100 ng/ml Dox, normalized for total protein concentration, and analyzed by SDS-PAGE/immunoblotting using antibodies for PCTAIRE1 (top) and beta-actin (bottom). (C, D) MDA-MB-468 cells were cultured with (ON) or without (OFF) 100 ng/ml Dox for 48 hours, then stimulated with various concentrations of either anti-Fas antibody (CH-11) (C) or TRAIL (D). After 24 hours, cellular ATP levels were measured, and the data expressed as the ratio relative to cells cultured without anti-Fas or TRAIL (mean ± SD; n = 3). (E, F) Clonogenic survival assays. MDA-MB-468 cells stably containing inducible shRNAs (scramble, shRNA#1, shRNA#2) were cultured with (ON) or without (OFF) 100 ng/ml Dox for 48 hours, then stimulated with anti-Fas antibody (CH-11, 250 ng/ml) or TRAIL (100 ng/ml). Cells were cultured for 3 days before fixing and staining with 0.5% crystal violet dye. (G) MDA-MD-468 cells containing inducible PCTAIRE1 targeting shRNA vectors were cultured with or without Dox (100 ng/ml) for 48 hours, then stimulated with or without TRAIL (100 ng/ml) for 4 hours. Cell lysates were prepared, normalized for total protein content, and analyzed by immunoblotting using antibodies specific for either proteolytically cleaved caspase-8 (p43/41, top) or beta-actin (bottom).
(TIF)

S5 Fig Conditional knockdown of PCTAIRE1 restores sensitivity of Du145 cells to TRAIL. (A) Du145 cells were stably infected with two different PCTAIRE1-targeting (tet-inducible) shRNA lentiviruses. Protein lysates were generated from Du145 cells cultured for 48 hours with (ON) or without (OFF) 100 ng/ml doxycycline (Dox), normalized for total protein concentration, and analyzed by SDS-PAGE/immunoblotting using antibodies for PCTAIRE1 (top) and beta-actin (bottom). (B) Du145 cells were cultured with (ON) or without (OFF) 100 ng/ml Dox for 48 hours, then stimulated with various concentrations of TRAIL. After 24 hours, cellular ATP levels were measured, and the data expressed as a ratio relative to cells cultured without TRAIL (mean ± SD; n = 3). (C) Du145 cells containing inducible PCTAIRE1 targeting shRNA vectors were cultured with or without Dox (100 ng/ml) for 48 hours, then stimulated with or without TRAIL (100 ng/ml) for 4 hours. Cell lysates were prepared, normalized for total protein content, and analyzed by immunoblotting using antibodies specific for either proteolytically cleaved caspase-8 (top, middle) or actin (bottom). The cleaved p43/41 and p18 bands of caspase-8 are indicated by arrows.
(TIF)

S6 Fig PCTAIRE1-knockdown does not affect the expression levels of various proteins related to the extrinsic pathway for apoptosis. (A) PPC-1 cells were transfected with control RNA or three different siRNAs targeting PCTAIRE1. After 48 hours, cell lysates were prepared, normalized for total protein content, and analyzed by immunoblotting using antibodies against various proteins related to the extrinsic pathway for apoptosis. The red arrow indicates non-specific bands. (B) Cell surface Fas, DR4 and DR5 expression was measured by flow cytometry of PPC1 cells transfected with scramble or siRNA targeting PCTAIRE1 (si-1472). Positive rates were assessed by comparison with IgG isotype control (data not shown). The data are representative of two independent experiments. (C, D) PPC1 cells were transfected with plasmids producing HA-tagged caspase-8, Myc-tagged PCTAIRE1 (wild type or kinase dead mutant), empty vector, or siRNAs (10 μM, scramble or si-1472). Lysates were either loaded directly onto gels (“Input”) or subjected to immunoprecipitation (IP) using anti-HA antibody. Immune complexes were analyzed by SDS–PAGE/immunoblotting. (E) PPC1 cells were transfected with plasmids producing HA-tagged FADD, Myc-tagged PCTAIRE1 (wild type or kinase dead mutant). Lysates were either loaded directly onto gels (“Input”) or subjected to immunoprecipitation (IP) using anti-HA antibody. (F) PPC1 cells were transfected with siRNAs (10μM, scramble or si-1472). After 48 hours, cell lysates were prepared, normalized for total protein content, and analyzed by immunoblotting.
(TIF)

S7 Fig PCTAIRE1 knockdown modulates RIPK1 expression in cancer cells. (A, C, E) PPC1, MDA-MB-468 and MCF7 cells were transfected with scrambled RNA or three different siRNA targeting PCTAIRE1 (siRNAs 1472, 1566, 1656). After 48 hours, relative levels of RIPK1 mRNA were measured by q-RT-PCR analysis. (B, D) MDA-MB-468 and MCF7 cells were transfected with siRNAs as indicated. Cell lysates 48 hours after transfection with siRNAs were prepared and analyzed by immunoblotting using mouse anti-RIPK1 (top) or anti-actin (bottom) antibodies. (F) HeLa cells were synchronized by a double thymidine block. Cell lysates were subjected to immunoblot analysis with indicated antibodies. (G) PPC1 cells were pretreated with the RIPK1 inhibitor necrostatin-1 (0, 5, 10, 50, 100 μM) for 1 hour, and then cells were treated with TRAIL (10 or 100 ng/ml). Cell viability was quantified by Cell Titer Glo after 24 hours. (H) PPC1 cells were transfected with siRNAs as indicated. Cell lysates 48 hours after transfection with siRNAs were prepared and analyzed by phos-tag SDS-PAGE/immunoblotting.
(TIF)

S8 Fig Knockdown of p27 does not restore Fas/TRAIL-resistance in PCTAIRE1-knockdown PPC1 cells. (A-C) PPC1 cells stably containing shRNA (#2) were reverse-transfected with 5 nM scramble-control RNA or siRNA targeting p27. After 8 hours, the culture media was changed to media with (Tet-ON) or without (Tet-OFF) 100 ng/ml doxycycline (Dox). After 48 hours, cell lysates were prepared, normalized for total protein content, and analyzed by immunoblotting using antibodies for PCTAIRE1 (top), p27 (middle) and actin (bottom) (A). (B, C) PPC1 cells were stimulated with either anti-Fas antibody (B) or TRAIL (C). After 24 hours, cellular ATP levels were measured using Cell Titer Glo reagents, with the data expressed as a ratio between cells cultured with and without anti-Fas (B) or TRAIL (C) (mean ± SD; n = 3). (D, E) PPC1 cells stably overexpressing p27 (wild type, S10A, T187A) were established by lentivirus infection and then stimulated with TRAIL. After 24 hours, cellular ATP levels were measured (mean ± SD; n = 3).
(TIF)
