# Supplementary figures and images for: PCTAIRE1-Knockdown Sensitizes Cancer Cells to TNF Family Cytokines
Source: PLoS One. 2015 Mar 19;10(3):e0119404. doi: 10.1371/journal.pone.0119404 (PMC4366397; doi:10.1371/journal.pone.0119404)

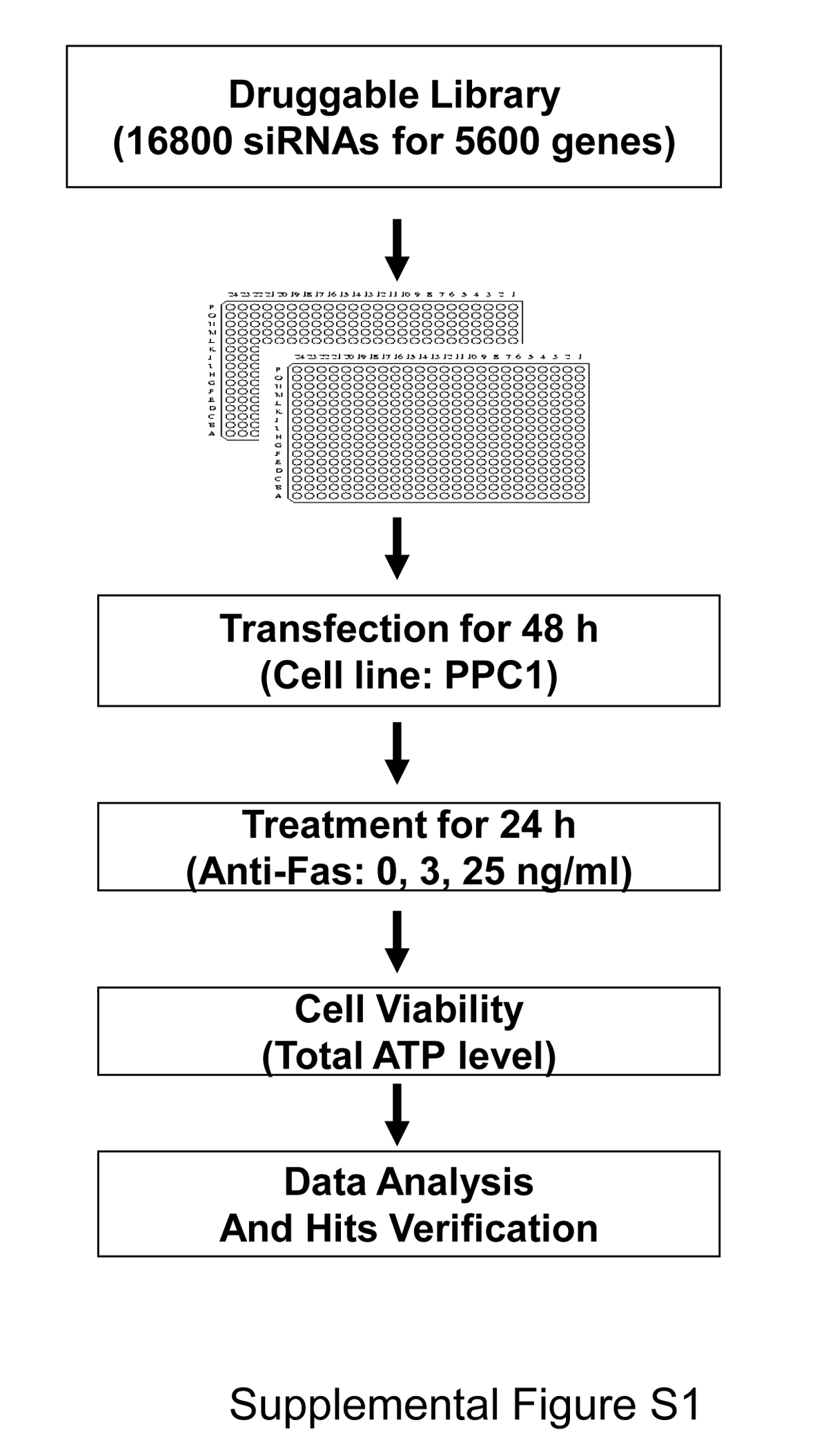

Supplement: S1 Fig — Two parallel HTS campaigns were conducted using low-dose (3 ng/ml) and high-dose (25 ng/ml) anti-Fas CH11 antibody. A library of 16,800 siRNAs constituting the “druggable” genome of 5,600 targets at 3-fold coverage was screened. (TIF) [file pone.0119404.s002.tif]

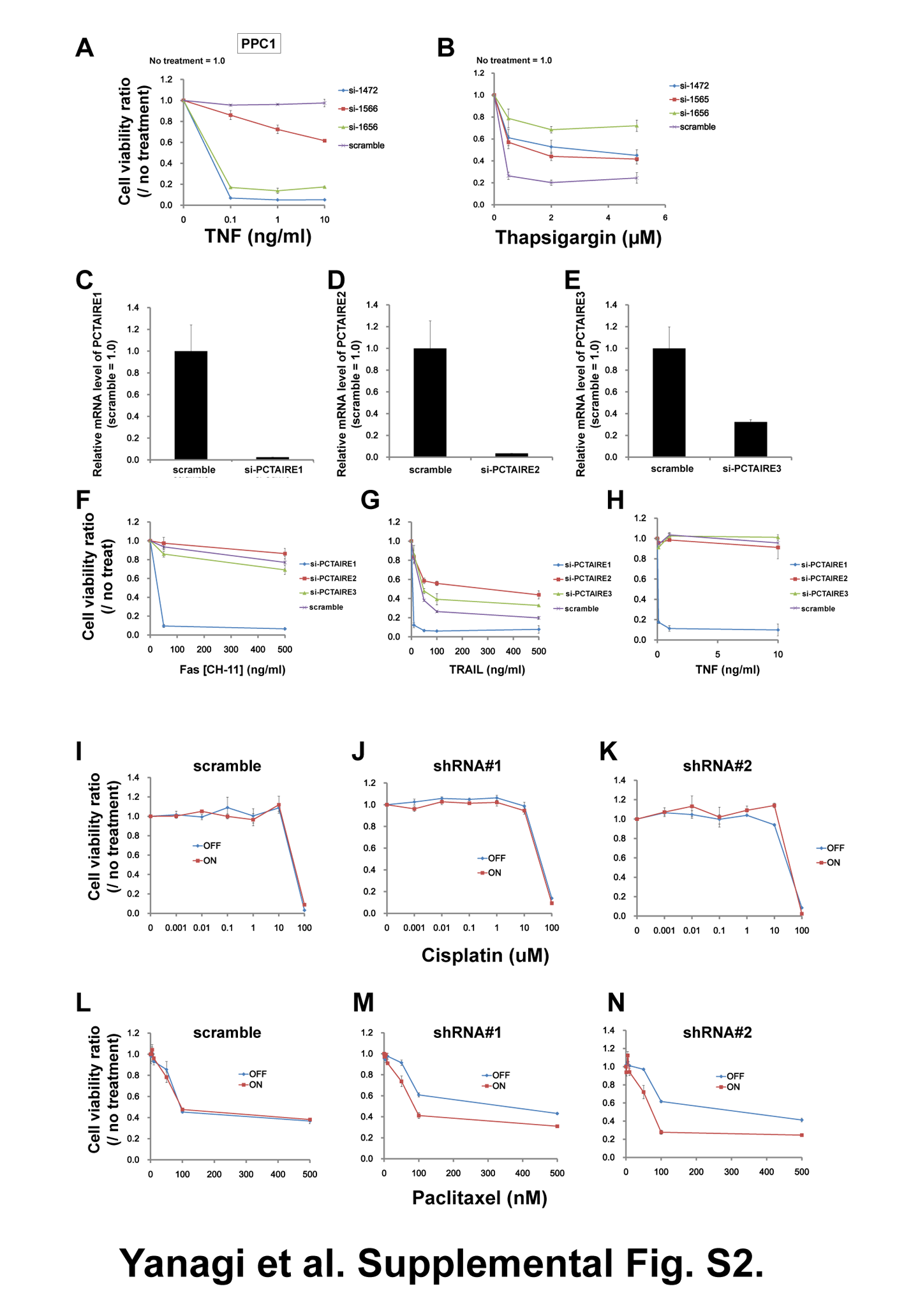

Supplement: S2 Fig — (A, B) PPC1 cells were transfected with control RNA (purple “x”) or various siRNAs targeting PCTAIRE1 (blue diamonds, 1472; red squares, 1566, green triangles, 1656). After 48 hours, cells were stimulated with either TNF (A) or thapsigargin (B) at the indicated concentrations. After 24 hours, cellular ATP levels were measured and the data expressed as the ratio between values for cells cultured with and without TNF (A) and thapsigargin (B) (mean ± SD; n = 3). (C-H) PPC1 cells were transfected with scrambled RNA or siRNAs targeting PCTAIRE1 (si-1472), PCTAIRE2 or PCTAIRE3. At 48 hours after transfection, mRNAs levels of PCTAIRE1 (C), PCTAIRE2 (D) and PCTAIRE3 (E) were measured by qRT-PCR, with normalization relative to GADPH (mean ± SD; n = 2). Forty-eight hours after transfection, cells were stimulated with Fas (CH-11) (F), TRAIL (G), or TNF (H) at various concentrations as indicated. After 24 hours, cellular ATP levels were measured, and the data expressed as the ratio between values for cells cultured with and without treatments (mean ± SD; n = 3). (I-N) PPC1 cells stably containing inducible shRNAs targeting different sites on PCTAIRE1 mRNA (shRNA#1, #2) or scramble-control were cultured for 48 hours with doxycycline (Dox, 100 ng/ml). PPC1 cells were cultured with (ON) or without (OFF) 100 ng/ml Dox for 48 hours, then stimulated with various concentrations of either cisplatin (I-K) or paclitaxel (L-N). After 24 hours, cellular ATP levels were measured and expressed as a ratio relative to cells cultured without treatments (mean ± SD; n = 3). (TIF) [file pone.0119404.s003.tif]

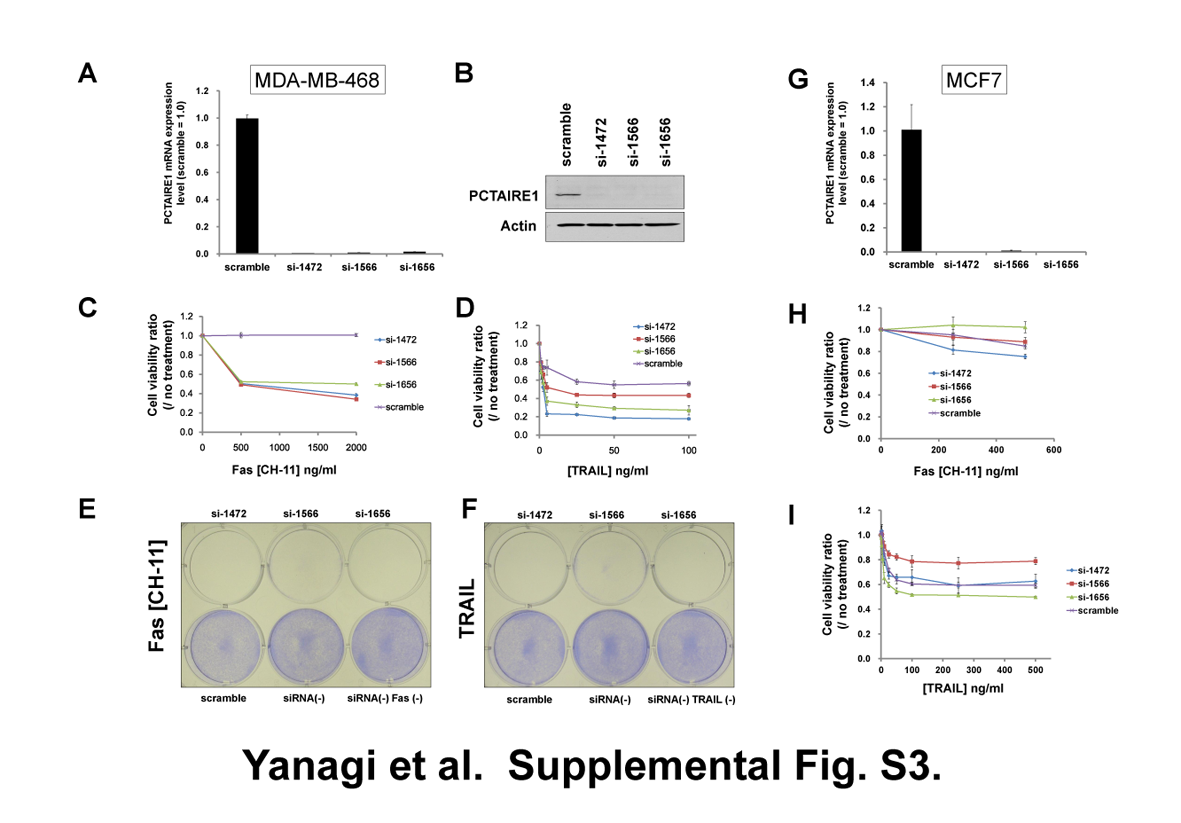

Supplement: S3 Fig — (A, G) MDA-MB-468 (A) and MCF7 (G) cells were transfected with scrambled RNA or three different siRNA targeting PCTAIRE1 (siRNAs 1472, 1566, 1656). After 48 hours, relative levels of PCTAIRE1 mRNA were measured by q-RT-PCR analysis. (B) Cell lysates 48 hours after transfection of siRNAs were prepared, normalized for total protein content, and aliquots were analyzed by immunoblotting using mouse anti-PCTAIRE1 (top) or anti-beta-actin (bottom) antibodies. (C, D, H, I) MDA-MB-468 (C, D) and MCF7 (H, I) cells were transfected with control RNA (purple “x”) or various siRNAs targeting PCTAIRE1 (blue diamonds, 1472; red squares, 1566; green triangles, 1656). After 48 hours, cells were stimulated with either anti-Fas antibody or TRAIL at various concentrations as indicated. After 24 hours, cellular ATP levels were measured and the data expressed as the ratio between values for cells cultured with and without anti-Fas (C, H) or TRAIL (D, I). All data represent mean ± SD (n = 3). (E, F) Clonogenic survival assays show that PCTAIRE1-knockdown sensitizes MDA-MB-468 cells to Fas and TRAIL. MDA-MB-468 cells were seeded at 2.0 x 10^5^ cells per well in 6 well (35 mm) dishes, then reverse-transfected with control or PCTAIRE1-targeting siRNAs as indicated. After 48 hours, anti-Fas antibody (250 ng/ml) (E) or TRAIL (100 ng/ml) (F) was added and cells were cultured for 72 hours before fixing and staining with 0.5% crystal violet dye. (TIF) [file pone.0119404.s004.tif]

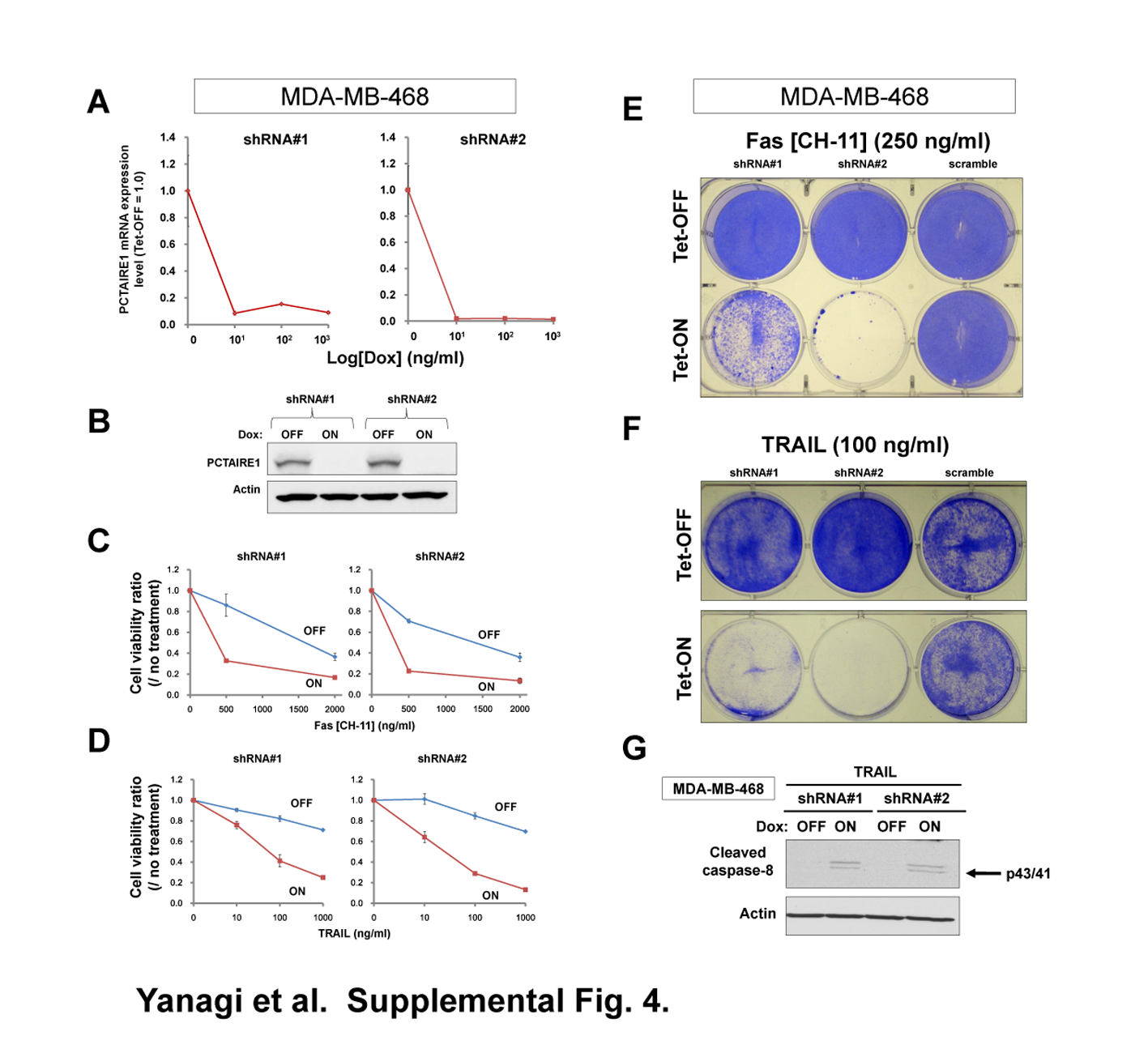

Supplement: S4 Fig — (A) MDA-MB-468 cells stably expressing inducible shRNAs targeting different sites on PCTAIRE1 mRNA (shRNA#1, #2) were cultured for 48 hours with various concentrations of doxycycline (Dox) ranging from 10 to 1000 ng/ml. PCTAIRE1 mRNA levels were measured by qRT-PCR, with normalization relative to GADPH (mean ± SD; n = 2). (B) Protein lysates were generated from MDA-MB-468 cells cultured for 48 hours with (ON) or without (OFF) 100 ng/ml Dox, normalized for total protein concentration, and analyzed by SDS-PAGE/immunoblotting using antibodies for PCTAIRE1 (top) and beta-actin (bottom). (C, D) MDA-MB-468 cells were cultured with (ON) or without (OFF) 100 ng/ml Dox for 48 hours, then stimulated with various concentrations of either anti-Fas antibody (CH-11) (C) or TRAIL (D). After 24 hours, cellular ATP levels were measured, and the data expressed as the ratio relative to cells cultured without anti-Fas or TRAIL (mean ± SD; n = 3). (E, F) Clonogenic survival assays. MDA-MB-468 cells stably containing inducible shRNAs (scramble, shRNA#1, shRNA#2) were cultured with (ON) or without (OFF) 100 ng/ml Dox for 48 hours, then stimulated with anti-Fas antibody (CH-11, 250 ng/ml) or TRAIL (100 ng/ml). Cells were cultured for 3 days before fixing and staining with 0.5% crystal violet dye. (G) MDA-MD-468 cells containing inducible PCTAIRE1 targeting shRNA vectors were cultured with or without Dox (100 ng/ml) for 48 hours, then stimulated with or without TRAIL (100 ng/ml) for 4 hours. Cell lysates were prepared, normalized for total protein content, and analyzed by immunoblotting using antibodies specific for either proteolytically cleaved caspase-8 (p43/41, top) or beta-actin (bottom). (TIF) [file pone.0119404.s005.tif]

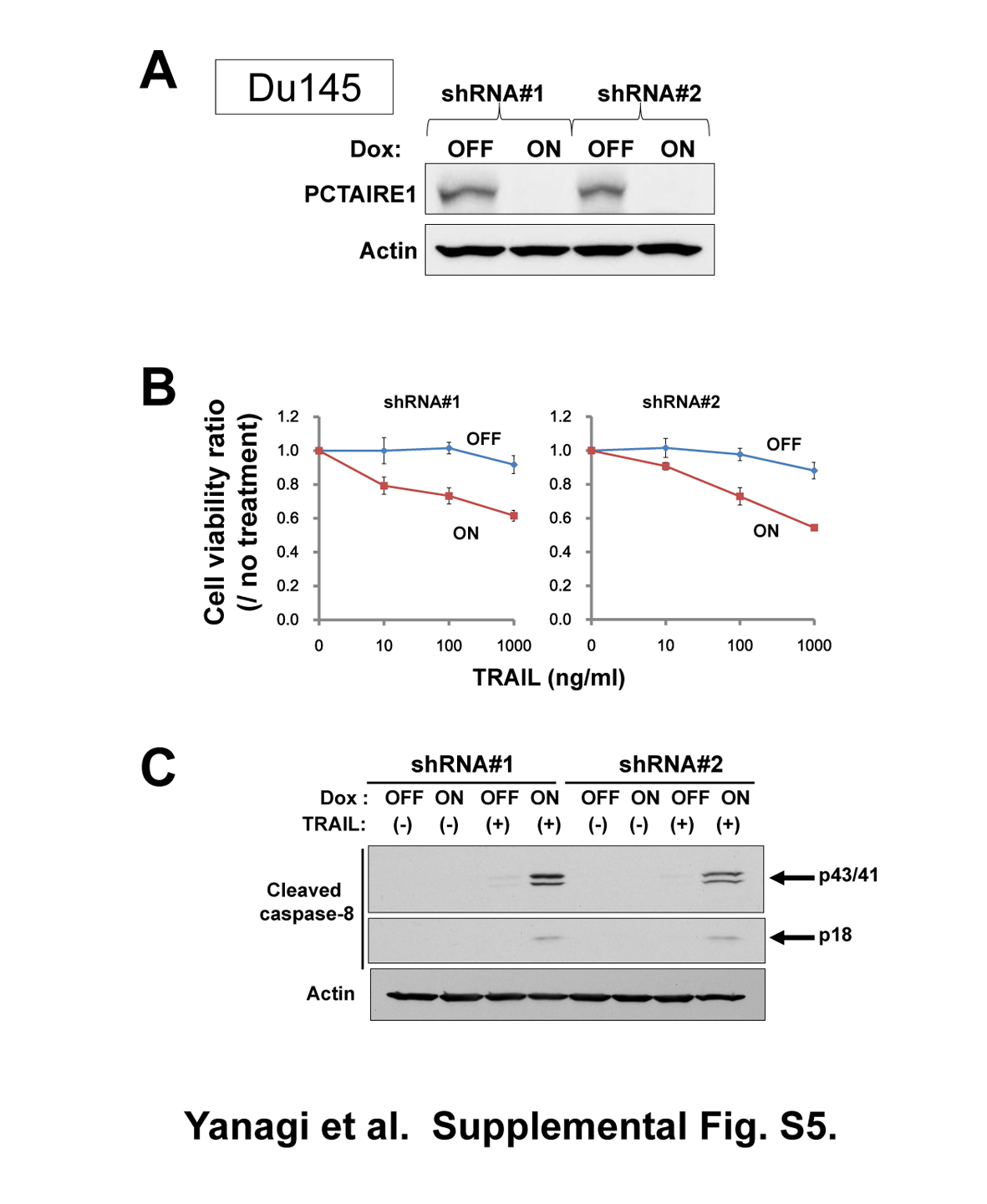

Supplement: S5 Fig — (A) Du145 cells were stably infected with two different PCTAIRE1-targeting (tet-inducible) shRNA lentiviruses. Protein lysates were generated from Du145 cells cultured for 48 hours with (ON) or without (OFF) 100 ng/ml doxycycline (Dox), normalized for total protein concentration, and analyzed by SDS-PAGE/immunoblotting using antibodies for PCTAIRE1 (top) and beta-actin (bottom). (B) Du145 cells were cultured with (ON) or without (OFF) 100 ng/ml Dox for 48 hours, then stimulated with various concentrations of TRAIL. After 24 hours, cellular ATP levels were measured, and the data expressed as a ratio relative to cells cultured without TRAIL (mean ± SD; n = 3). (C) Du145 cells containing inducible PCTAIRE1 targeting shRNA vectors were cultured with or without Dox (100 ng/ml) for 48 hours, then stimulated with or without TRAIL (100 ng/ml) for 4 hours. Cell lysates were prepared, normalized for total protein content, and analyzed by immunoblotting using antibodies specific for either proteolytically cleaved caspase-8 (top, middle) or actin (bottom). The cleaved p43/41 and p18 bands of caspase-8 are indicated by arrows. (TIF) [file pone.0119404.s006.tif]

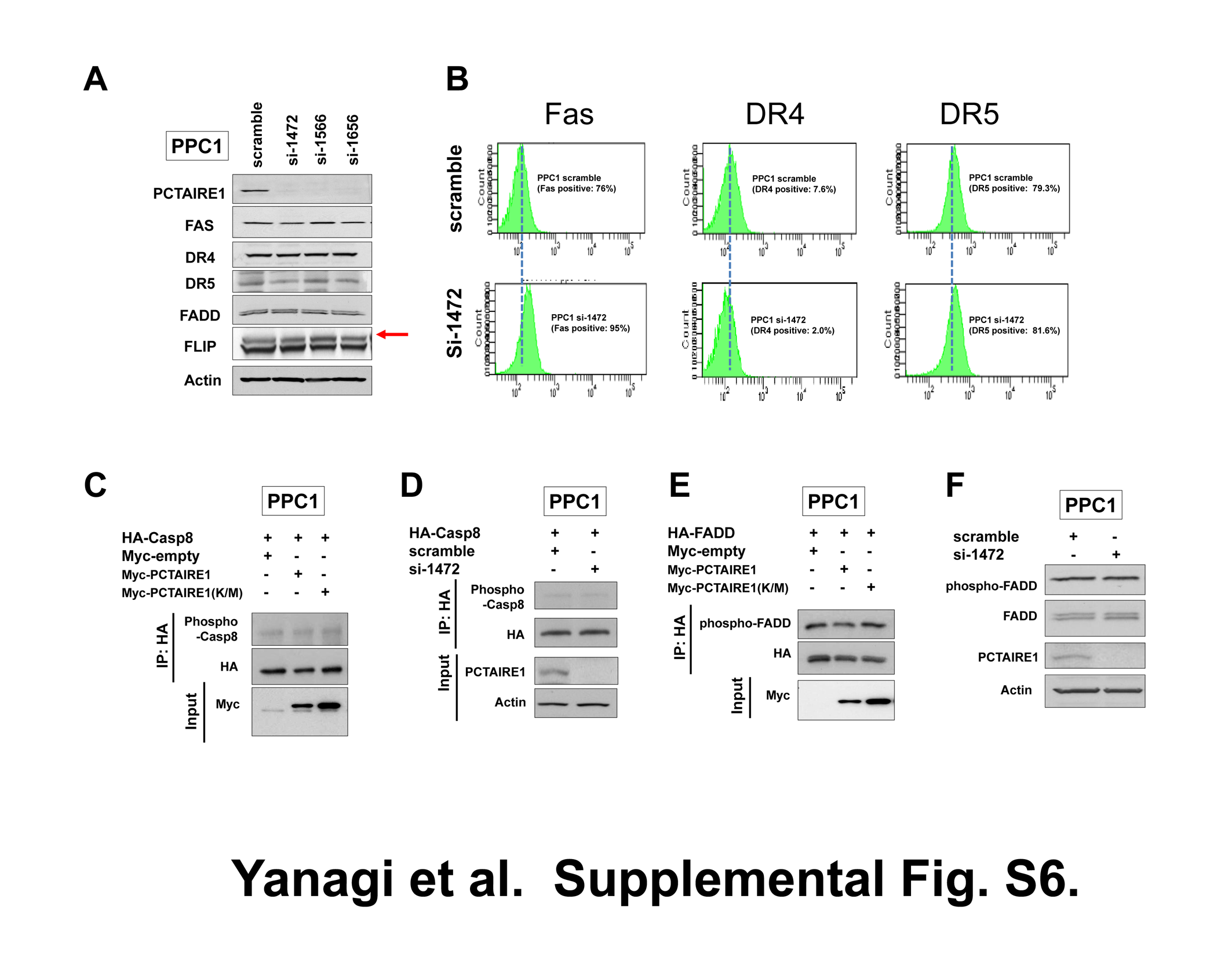

Supplement: S6 Fig — (A) PPC-1 cells were transfected with control RNA or three different siRNAs targeting PCTAIRE1. After 48 hours, cell lysates were prepared, normalized for total protein content, and analyzed by immunoblotting using antibodies against various proteins related to the extrinsic pathway for apoptosis. The red arrow indicates non-specific bands. (B) Cell surface Fas, DR4 and DR5 expression was measured by flow cytometry of PPC1 cells transfected with scramble or siRNA targeting PCTAIRE1 (si-1472). Positive rates were assessed by comparison with IgG isotype control (data not shown). The data are representative of two independent experiments. (C, D) PPC1 cells were transfected with plasmids producing HA-tagged caspase-8, Myc-tagged PCTAIRE1 (wild type or kinase dead mutant), empty vector, or siRNAs (10 μM, scramble or si-1472). Lysates were either loaded directly onto gels (“Input”) or subjected to immunoprecipitation (IP) using anti-HA antibody. Immune complexes were analyzed by SDS–PAGE/immunoblotting. (E) PPC1 cells were transfected with plasmids producing HA-tagged FADD, Myc-tagged PCTAIRE1 (wild type or kinase dead mutant). Lysates were either loaded directly onto gels (“Input”) or subjected to immunoprecipitation (IP) using anti-HA antibody. (F) PPC1 cells were transfected with siRNAs (10μM, scramble or si-1472). After 48 hours, cell lysates were prepared, normalized for total protein content, and analyzed by immunoblotting. (TIF) [file pone.0119404.s007.tif]

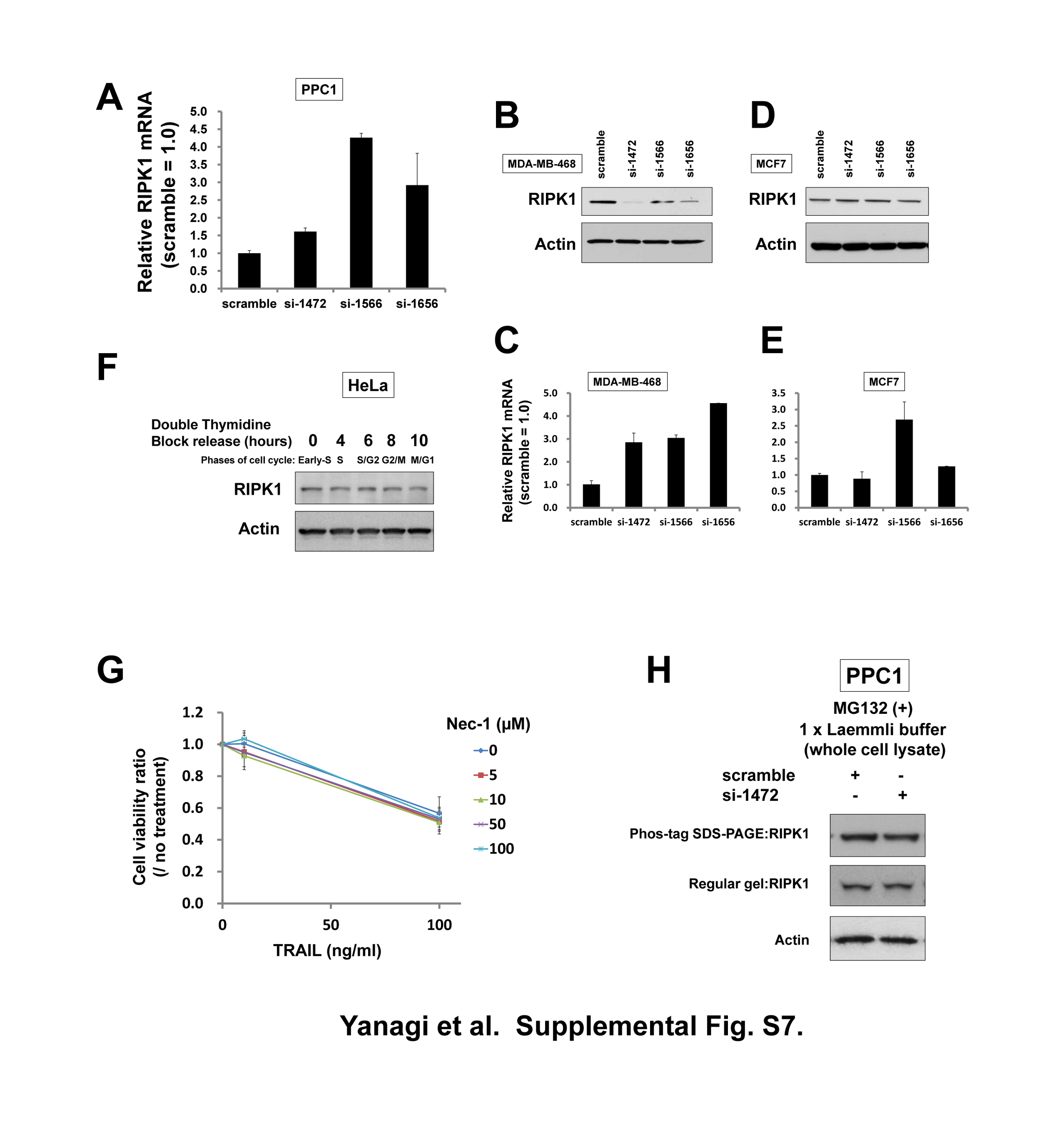

Supplement: S7 Fig — (A, C, E) PPC1, MDA-MB-468 and MCF7 cells were transfected with scrambled RNA or three different siRNA targeting PCTAIRE1 (siRNAs 1472, 1566, 1656). After 48 hours, relative levels of RIPK1 mRNA were measured by q-RT-PCR analysis. (B, D) MDA-MB-468 and MCF7 cells were transfected with siRNAs as indicated. Cell lysates 48 hours after transfection with siRNAs were prepared and analyzed by immunoblotting using mouse anti-RIPK1 (top) or anti-actin (bottom) antibodies. (F) HeLa cells were synchronized by a double thymidine block. Cell lysates were subjected to immunoblot analysis with indicated antibodies. (G) PPC1 cells were pretreated with the RIPK1 inhibitor necrostatin-1 (0, 5, 10, 50, 100 μM) for 1 hour, and then cells were treated with TRAIL (10 or 100 ng/ml). Cell viability was quantified by Cell Titer Glo after 24 hours. (H) PPC1 cells were transfected with siRNAs as indicated. Cell lysates 48 hours after transfection with siRNAs were prepared and analyzed by phos-tag SDS-PAGE/immunoblotting. (TIF) [file pone.0119404.s008.tif]

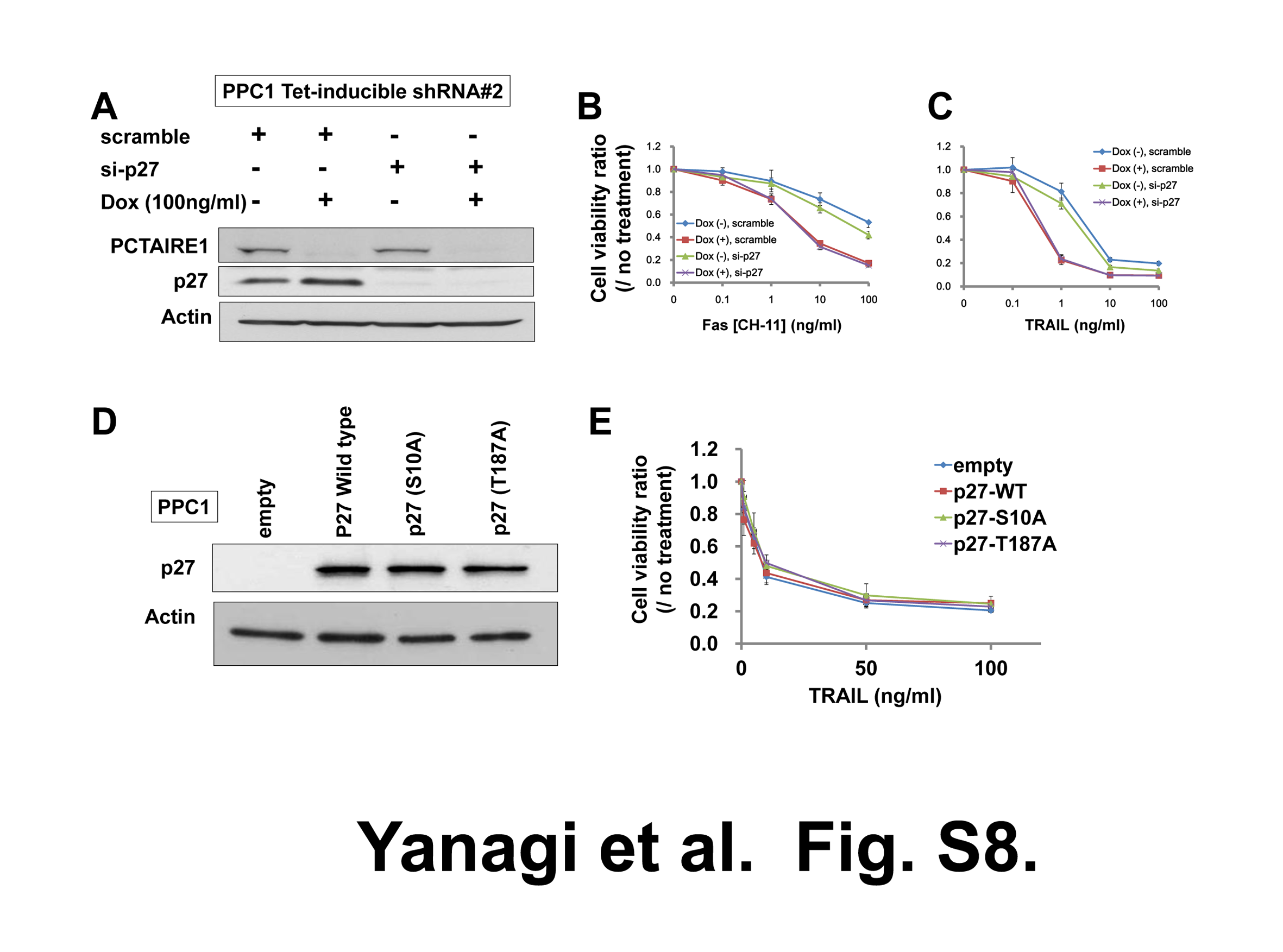

Supplement: S8 Fig — (A-C) PPC1 cells stably containing shRNA (#2) were reverse-transfected with 5 nM scramble-control RNA or siRNA targeting p27. After 8 hours, the culture media was changed to media with (Tet-ON) or without (Tet-OFF) 100 ng/ml doxycycline (Dox). After 48 hours, cell lysates were prepared, normalized for total protein content, and analyzed by immunoblotting using antibodies for PCTAIRE1 (top), p27 (middle) and actin (bottom) (A). (B, C) PPC1 cells were stimulated with either anti-Fas antibody (B) or TRAIL (C). After 24 hours, cellular ATP levels were measured using Cell Titer Glo reagents, with the data expressed as a ratio between cells cultured with and without anti-Fas (B) or TRAIL (C) (mean ± SD; n = 3). (D, E) PPC1 cells stably overexpressing p27 (wild type, S10A, T187A) were established by lentivirus infection and then stimulated with TRAIL. After 24 hours, cellular ATP levels were measured (mean ± SD; n = 3). (TIF) [file pone.0119404.s009.tif]
